# Willingness and attitudes of the general public towards the involvement of medical students in their healthcare

**Published:** 2012-09-30

**Authors:** Mariam Abu Jubain, Hajar Alobaidi, Sanah Bholah, Farah Kanani, Raveen Koghar, Hannah Shereef, Alice Sitch

**Affiliations:** 1 College of Medical and Dental Sciences, University of Birmingham, UK; 2 Public Health, Epidemiology and Biostatistics, School of Health and Population Sciences, College of Medical and Dental Sciences, University of Birmingham, UK

## Abstract

**Objectives:**

To determine if patients allow medical students to perform less invasive procedures compared to more invasive procedures, and how this is related to patient demographics and previous experience with medical students.

**Methods:**

A cross-sectional survey was conducted in six areas of Birmingham, UK. All members of the general public over the age of 18 were eligible, excluding non-English speaking people and those with cognitive impairments. Respondents were asked to rank their willingness for medical students to perform history taking/examinations and clinical procedures of varying degrees of invasiveness.

**Results:**

We received a total of 293 responses. For both history taking/examinations and clinical procedures, people were more willing to allow medical students to perform less invasive procedures rather than more invasive procedures. White and older people were more willing to allow all history taking/examinations procedures; additionally, women were more willing to allow history taking. White, female, and older participants were more willing to allow blood pressure measurement; whilst older people and those with previous experience were more willing to allow venepuncture. No significant associations were found for intubation.

**Conclusions:**

The public is less willing for medical students to perform more invasive procedures. This may severely limit opportunities to attain clinical competencies.

## Introduction

Learning in the clinical setting forms an integral part of undergraduate medical education. UK General Medical Council (GMC) guidelines, detailed in Tomorrow’s Doctors,^1^ state that “students should have the opportunity to become increasingly competent in their clinical skills and in planning patient care,” and they “should have a defined role in medical teams, subject to considerations of patient safety.” The importance of obtaining informed consent has often been stressed, although in reality, some studies have shown that consent is not always fully informed.^2^,^3^ Little is known about the attitudes of patients in the National Health Service (NHS) to the involvement of medical students in different aspects of their healthcare.

Previous studies addressing the topic have focused on the presence of medical students in outpatient clinics,^4^,^5^ in hospital wards,^6^ or during bedside teaching.^7^ Other studies have looked at patient willingness in specific medical departments.^5^,^8^–^10^ Some have reported decreased rates of patient willingness in obstetrics and gynaecology and genitourinary specialities.^11^,^12^ It is suggested that this may be because of the more invasive and intimate nature of the examinations. Although these studies provide an insight into patient attitudes to passive medical student involvement, that is, simply *observing* patient interactions with qualified doctors, they may have differing views towards active medical student involvement in their healthcare, that is, the *performing* of examinations or clinical procedures by students themselves, under the supervision of qualified professionals. Few studies have looked at whether there are any differences in willingness between a student performing a more invasive examination e.g. rectal or vaginal, compared to a less invasive examination e.g. abdominal.

Similarly, not many studies^13^–^15^ have investigated patients’ attitudes towards medical students performing different clinical procedures, such as venepuncture. Graber et al.^13^ asked 150 patients for their opinions regarding medical students carrying out procedures as part of their training. They found that patient willingness was low for highly invasive procedures. This study, however, was based on a small population sample in an emergency department in the USA and it is not known whether the results would be reproducible in the UK.

Additionally, few studies have examined the demographic characteristics of patients who may be unwilling to allow a medical student to participate in their healthcare. Results across the few existing studies investigating the effect of the patients’ age on willingness are inconsistent, and most were conducted in a clinical setting with small sample sizes for younger age groups that may not be representative of the views of the general population.^5^,^13^ With regard to ethnicity, a US study by Adams et al.^16^ showed that non-White patients in an ambulatory care setting were less likely to perceive a benefit from the participation of medical students in their care compared to White patients. Non-White respondents also expressed greater concern if they were examined by a medical student alone, without supervision from a qualified professional. It would be important to conduct the study in large cities, such as Birmingham, which has one of the most ethnically diverse populations in the UK.^17^ This would allow large enough sample sizes to be obtained so that we could analyse, not only whether there is a difference between White people and ethnic minorities in the UK in willingness to allow active medical student participation in their care, but also whether there are any differences in willingness between different ethnic minority groups. A study by Simons et al.^5^ has suggested that previous contact with medical students in a clinical setting may increase patient willingness for subsequent medical student participation in their care. However, since this study was conducted in just one general internal medicine practice, it is unknown if this association was specific to this institution or whether it can be extended to patients elsewhere.

### Aims and objectives

We investigated the perceptions of members of the general population, who may access healthcare from the NHS, regarding the active involvement of medical students in their healthcare. This study builds on existing research by sampling the general population, rather than a clinical population, and determining if people with specific demographic characteristics (e.g. age, sex, and ethnicity) report different levels of comfort with medical students performing invasive procedures. This study will highlight the relative importance of patient attitudes as a factor influencing opportunities for medical students to attain a full range of clinical competencies.

## Methods

### Study design and population

We conducted a cross-sectional survey of members of the general public in Birmingham, UK, who can potentially receive healthcare from the NHS. The study was conducted across six areas of Birmingham, with varying socioeconomic populations.^17^ Systematic sampling was used to approach every fifth person who passed a well-known landmark in each area. All members of the general public over the age of 18 years were eligible, with the exception of non-English speaking people and those with cognitive impairments. Upon approaching members of the public, the purpose of the survey was explained, verbal consent was requested, and a self-completion questionnaire was given to the participant. A total of 293 people agreed to complete the survey, which represents about 80% of those who were asked to participate, although this varied considerably from 60% to 95%, depending on the area sampled.

### Clinical procedures

The questionnaire consisted of six questions, with the final question comprising six sub-parts. The first three items were demographic questions about sex, age and ethnicity of the participant. Age was represented by three categories, 18–40 years, 41–64 years, and 65+ years; and ethnicity was indicated as Black, Chinese, South Asian, White, Mixed, or Other. The next two questions asked whether the participant had encountered medical students in a general practice, a hospital or other healthcare setting. The final question asked participants to rate their willingness to allow medical students, who had received simulation training, to perform six clinical procedures on them. Each procedure was briefly described.

Three of the clinical procedures involved history taking/examinations: taking a history, performing a less invasive physical examination (such as examining the abdomen), and performing a more invasive physical examination (such as a rectal examination). The other three were clinical procedures such as: measuring blood pressure, taking a blood sample, and inserting a tube (intubation). In each group, the procedures were presented in order of increasing level of invasiveness.

### Measures

The list of clinical competencies for medical students, detailed by the UK General Medical Council,^1^ was used as a guide to selecting clinical procedures for the questionnaire. Those procedures most frequently performed by healthcare professionals were chosen, as they would be most familiar to survey participants.

Willingness to allow medical students to perform each clinical procedure was assessed with Likert ratings: 1= not willing, 2 = not really willing, 3 = undecided, 4 = somewhat willing, and 5 = willing. Questionnaires were completed independently by the participants.

### Data analysis

The characteristics of the sample were tabulated. Due to small frequency counts for some ethnicity groups, Chinese, mixed, and other ethnicities were combined into one group. South Asian and Black ethnicities formed two separate groups, as these represent the largest ethnic minority groups in Birmingham, according to the 2001 Population Census.^17^

The willingness to participate in each procedure was assessed by investigating the proportion of people reporting the ‘willing’ option. This was then further investigated by calculating a mean willingness score for each skill and using regression models to calculate the difference in willingness score as the skill became more invasive. Modelling was done separately for the history taking/examination and clinical procedures categories.

The willingness scores were then further analysed using analysis of variance (ANOVA) to determine differences in willingness scores across demographic characteristics.

## Results

### Characteristics of the sample

As shown in Table 1, a similar number of male and female participants responded to the survey and the majority was in the 18–40 year-old age group. With regards to ethnicity, most of our sample population were White, followed by South Asian and Black. More than half of the respondents had no previous experience with medical students. Of those who had, most encountered them in hospitals, followed by general practice settings, or both.

### Assessment of willingness by clinical skill

As shown in Table 2, the proportion of ‘willing’ responses declined as the procedures became more invasive. In the history taking/examinations group, participants were most willing to allow history taking, the least invasive procedure in the group, with the proportion of ‘willing’ responses at 0.67 (95% CI is 0.62 to 0.73). In contrast, the proportion of ‘willing’ responses for more invasive examination, the most invasive procedure in the group, was only 0.19 (95% CI is 0.15 to 0.24). For the clinical procedures, the proportion of ‘willing’ responses for the least invasive procedure, measuring blood pressure, was 0.78 (95% CI is 0.73 to 0.82) and for the most invasive procedure, intubation, it was lower at 0.14 (95% CI is 0.10 to 0.18).

In the history taking/examinations group, there was no significant difference in willingness between less invasive examination (moderately invasive) and history taking (least invasive). However, willingness for invasive examination (most invasive) was significantly lower when compared to history taking, with a difference of −1.61 (95% CI= (−1.75, −1.47); *p* < 0.001). In the clinical procedures group, there were significant decreases in willingness between measuring blood pressure (least invasive) and taking a blood sample (moderately invasive)(difference=−0.71; 95% CI=(−0.87, −0.55); *p* < 0.001), and tube insertion (most invasive) (difference=−2.12; 95% CI=(−2.28, −1.95); *p* <0.001).

### Associations between willingness, clinical skill and sex, age group, ethnicity, previous experience and experience setting

#### History taking/examinations

As shown in Table 3, willingness for history taking appears to be greater among women, older age groups and White respondents. Regarding less invasive examination, willingness was higher for older age groups and was lower in ethnic minority groups compared to Whites. The older age groups, White respondents, and respondents who had previously interacted with medical students in both hospital and general practice settings were most willing to have medical students perform a more invasive examination.

#### Clinical procedures

As shown in Table 3, women were more willing to have their blood pressure measured than men. Willingness for blood pressure measurement also increased with age group and was greatest for White respondents. For venepuncture, willingness increased with age and previous experience. In contrast, willingness for medical students to perform intubation was not found to vary significantly with sex, age, ethnicity, previous experience, or medical setting.

## Discussion

Our results clearly show that, as the invasiveness of the procedure increases, the rates of willingness of the general public to allow medical students to perform the procedure decreases, ranging from about two-thirds willing to allow history taking to a fifth for invasive examinations in the history taking/examinations group; and about three quarters willing to allow blood pressure measurement to under a fifth for tube insertion in the clinical procedures group. Our findings have been supported by similar studies.^13^,^18^ This is understandable, given that more invasive procedures may be uncomfortable and perceived as being more dangerous or having longer lasting side effects.

Willingness was higher for measuring blood pressure than for history taking and for less invasive examination compared to taking a blood sample. This indicates that people are more willing for medical students to perform a minimally invasive procedure than take a history, which may involve answering questions about their personal and social lives. It may also suggest that people perceive blood pressure measurement to be a low-risk procedure. However, people are less willing to allow venepuncture, the next most invasive clinical procedure, than to allow a less invasive examination to be performed, possibly because they foresee a greater level of risk in the former.

In terms of differences between men and women, we found that women were more willing than men to allow history taking and blood pressure measurement. Because women are more likely to seek help from health professionals during their lifetime,^19^ they may have greater familiarity with less invasive procedures, and thus be more open to a medical student performing them. This is in contrast to previous studies which have indicated that men are generally more accepting of medical student participation in their healthcare.^5^

Regarding age, there was a significant difference in willingness across age groups for all procedures (except intubation), with the largest difference seen for more invasive examination. Other studies have shown variable results when investigating the effect of age on willingness.^5^,^13^ However, these studies were conducted in the clinical setting, and therefore may have lower numbers of young respondents. Older people are more likely to have received care within the NHS, and thus may be more willing to participate in medical education, as their way of ‘giving back’. They may also have previous experience of a procedure and may be less concerned about side effects, for example bruising after venepuncture, which may be perceived as an unacceptable complication in a younger patient.

With regard to ethnicity, willingness was higher in Whites compared to non-Whites for all procedures except taking a blood sample and intubation. The decreased willingness among ethnic minorities is reflected by similar findings in previous research. Adams et al.^16^ showed that, in a general medicine clinic, non-White patients were less comfortable with medical student involvement in their care compared to White patients. This may be attributed to language or communication difficulties should complications arise during the procedure. Cultural and religious barriers have also been suggested to play a role, for example decreased willingness among Muslim women to allow a male medical student to participate in their care.^20^

The only procedure for which respondents with previous experience of medical students had a higher proportion of willingness was taking a blood sample. However, previous studies have shown a positive influence of previous encounters on levels of willingness for medical students to participate in their healthcare.^5^ This may be because patients with prior experience are more familiar with the role of medical students in healthcare. However, patients with previous experience are also more likely to be older or have had greater contact with the NHS, and therefore may be grateful for an opportunity to ‘give back’ to the healthcare system. This could, therefore, act as a confounding factor.

### Limitations

We used street surveys to obtain our sample population, which may have introduced sampling bias as our allocation of questionnaires was not randomised. Responder bias may also have affected our sample population as those individuals who did respond to our questionnaire may be inherently different from non-responders. The exclusion of non-English speaking and cognitively impaired subjects may have reduced the generalizability of our study since they are also likely to interact with medical students in the NHS.

We attempted to limit the extent of interviewer bias by providing no additional information regarding the content of the questionnaire. However, as medical students, we may have introduced accountability bias. Non-authority figures outside of the medical profession could have conducted the survey to eliminate this bias.

A potential confounding factor may be that we did not account for other demographic variables of each demographic sub-group; for example the results from the 18–40 age group may have been due to most members of that group being of a particular ethnicity or sex, rather than because of their age. Further unaccounted confounding factors include current health status and socioeconomic class of respondents. We attempted to eliminate the influence of socioeconomic class on our results by conducting the survey in areas of Birmingham with varying demographics and during different hours of the day to enable us to sample the working population.

Although the study was conducted outside the clinical environment, the results may not be fully representative of the views of patients in a clinical setting, as attitudes may change when directly faced with ill-health and the imminent prospect of the procedure in question. Furthermore, our literature search revealed other factors that may be important, such as characteristics of the medical student performing the procedure, including age, sex, appearance, and previous experience and training.^14^,^16^,^18^,^20^ However, a highly extensive study would be required to analyse all these potential variables.

### Implications and recommendations

The low numbers of people who are willing for medical students to perform more invasive examinations and procedures pose a significant problem for medical education, as opportunities to practise these skills may be severely limited.

It is important to alleviate fear among patients and increase levels of willingness among them. This may be in the form of proper explanations of the role of medical students as doctors in training,^18^ assurances of the presence of a fully qualified supervisor,^21^ and assurances of prior training in a simulated setting.^14^ Studies have also shown that prior notice of medical student involvement may be appreciated among patients.^21^ Finally, communication skills training should form an essential part of the undergraduate curriculum for medical students, providing guidance on how to introduce themselves to patients, explain their role in the clinical setting, obtain informed consent and put the patient at ease throughout the procedure.

### Conclusions

Our study found that the majority of our sample population from Birmingham show willingness for medical students to perform non-invasive procedures such as history taking and measuring blood pressure. However, as the invasiveness of a procedure increases, there is a notable decrease in willingness of the general public for a medical student to be actively involved in their healthcare. We have shown that rates of willingness are influenced by patient demographics such as age, sex, ethnicity and previous experience.

The low levels of willingness for more invasive procedures has significant implications for medical students, as opportunities to attain these clinical competencies may be severely limited. It is clear that this is an issue that needs to be addressed to maintain a high standard of medical education whilst ensuring patient satisfaction in clinical settings.

## Figures and Tables

**Table 1:** Characteristics of the sample (*n* = 293)

Characteristic	Number (%)
***Sex***	
Male	138 (47.1)
Female	155 (52.9)
***Age Group (years)***	
18–40	166 (56.7)
41–64	71 (24.2)
65+	56 (19.1)
***Ethnicity***	
White	160 (54.6)
South Asian	64 (21.8)
Black	22 (7.5)
Chinese	9 (3.1)
Mixed	17 (5.8)
Other	21 (7.2)
***Previous Experience***	
None	152 (51.9)
Yes- Hospital	51 (17.4)
Yes- General Practice	38 (13.0)
Yes- Both Locations	34 (11.6)
Yes- Other Location	18 (6.1)

**Table 2:** Proportions and 95% confidence intervals (binomial exact) for ‘willing’ responses (5 on the Likert scale), mean willingness values for each procedure and the differences and *p*-values from the regression analysis (*n* = 293)

		Number	Proportion	95% Confidence Interval	Mean *(SD)*	Difference (95% CI)	*p*-value
	
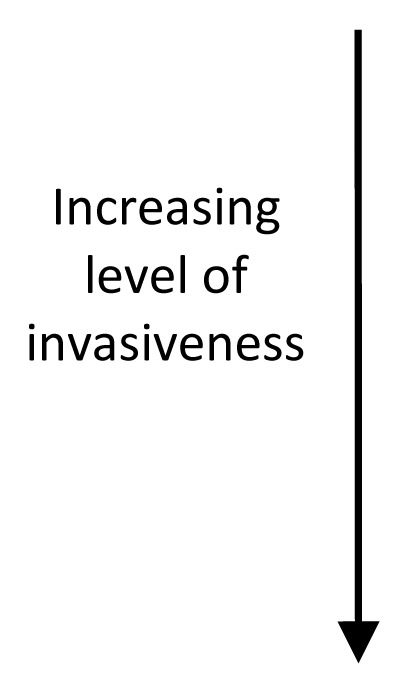	**History taking/examination**						
	History Taking (least invasive)	197	0.67	(0.62, 0.73)	4.46 (0.94)	-	-
	Less invasive Examination	175	0.60	(0.54, 0.65)	4.36 (0.98)	−0.11 (−0.25, 0.04)	0.143
	More invasive Examination (most invasive)	56	0.19	(0.15, 0.24)	2.85 (1.46)	−1.61 (−1.75, −1.47)	< 0.001
	**Clinical procedures**						
	Measuring Blood Pressure (least invasive)	228	0.78	(0.73, 0.82)	4.63 (0.84)	-	-
	Taking a Blood Sample	140	0.48	(0.42, 0.54)	3.92 (1.30)	−0.71 (−0.87, −0.55)	< 0.001
	Inserting Tubes (most invasive)	40	0.14	(0.10, 0.18)	2.52 (1.43)	−2.12 (−2.28, −1.95)	< 0.001

History taking is the reference procedure for History taking/Examination and Blood pressure is the reference procedure for clinical procedures. Wald statistic for both History taking/examination and Clinical procedures is < 0.001.

**Table 3:** Results of ANOVA to investigate differences in willingness for each procedure by sex, age, ethnicity, previous experience and setting of previous experience

	HISTORY TAKING/EXAMINATIONS (least invasive to most invasive)						CLINICAL PROCEDURES (least invasive to most invasive)					

	History Mean (*SD*) *p* value		Less invasive Examination Mean *(SD)*		More invasive Examination Mean *(SD)*		Blood Pressure Mean *(SD)*		Blood Sample Mean *(SD)*		Tubes Mean (SD)	

***Sex***												
Male	4.30 (1.04)		4.29 (0.98)		2.93 (1.45)		4.48 (1.03)		3.83 (1.30)		2.58 (1.49)	
Female	4.61 (0.82)	*p < 0.01*	4.42 (0.98)	*p = 0.259*	2.78 (1.46)	*p = 0.367*	4.77 (0.59)	*p < 0.01*	4.01 (1.29)	*p = 0.220*	2.46 (1.37)	*p = 0.492*
***Age Group***												
18–40 yrs	4.27 (1.05)		4.07 (1.11)		2.43 (1.34)		4.48 (0.98)		3.54 (1.38)		2.43 (1.37)	
41–64 yrs	4.68 (0.75)		4.65 (0.70)		3.38 (1.33)		4.80 (0.60)		4.38 (0.93)		2.59 (1.43)	
65+ yrs	4.78 (0.66)	*p < 0.001*	4.84 (0.42)	*p < 0.001*	3.45 (1.52)	*p < 0.001*	4.90 (0.45)	*p < 0.001*	4.50 (1.03)	*p < 0.001*	2.68 (1.60)	*p = 0.481*
***Ethnicity***												
Black	4.50 (0.86)		4.14 (1.32)		2.68 (1.52)		4.32 (1.17)		3.82 (1.62)		2.41 (1.62)	
South Asian	4.13 (1.29)		3.73 (1.34)		2.33 (1.33)		4.42 (1.17)		3.63 (1.32)		2.19 (1.42)	
White	4.66 (0.68)		4.69 (0.55)		3.18 (1.46)		4.81 (0.54)		4.13 (1.25)		2.68 (1.43)	
Other	4.23 (1.03)	*p < 0.01*	4.17 (0.89)	*p < 0.001*	2.53 (1.32)	*p < 0.001*	4.49 (0.86)	*p < 0.01*	3.70 (1.18)	*p = 0.106*	2.49 (1.32)	*p = 0.289*
***Previous Experience***												
Yes	4.54 (0.88)		4.46 (0.96)		2.90 (1.49)		4.71 (0.76)		4.11 (1.21)		2.55 (1.43)	
No	4.40 (0.99)	*p = 0.214*	4.26 (0.99)	*p = 0.076*	2.81 (1.43)	*p = 0.600*	4.56 (0.90)	*p = 0.121*	3.75 (1.35)	*p = 0.05*	2.49 (1.43)	*p = 0.721*
***Setting***												
Hospital	4.41 (0.94)		4.37 (1.06)		2.76 (1.49)		4.51 (1.07)		4.24 (1.07)		2.57 (1.47)	
General Practice	4.63 (0.88)		4.47 (1.06)		3.03 (1.46)		4.92 (0.27)		4.32 (1.04)		2.39 (1.26)	
Both	4.50 (0.90)		4.50 (0.90)		3.47 (1.44)		4.73 (0.67)		4.06 (1.39)		2.97 (1.62)	
Other	4.67 (0.77)	*p = 0.609*	4.56 (0.51)	*p = 0.884*	1.94 (1.11)	*p < 0.01*	4.83 (0.38)	*p = 0.071*	3.50 (1.42)	*p = 0.098*	1.94 (1.06)	*p = 0.084*
